# Evaluating the impact of different deface algorithms on deep learning segmentation software performance

**DOI:** 10.3389/fonc.2025.1603593

**Published:** 2025-10-07

**Authors:** Ali Ammar, Libing Zhu, Shep Bryan, Nathan Y. Yu, Carlos Vargas, Yi Rong, Quan Chen

**Affiliations:** Department of Radiation Oncology, Mayo Clinic, Phoenix, AZ, United States

**Keywords:** segmentation, defacing, computed tomography (CT), *mri_reface*, DeIdentifier, CARINA AI, radiation therapy, head and neck

## Abstract

**Introduction:**

Data sharing is essential for advancing research in radiation oncology, particularly for training artificial intelligence (AI) models in medical imaging. However, privacy concerns necessitate de-identification of medical images, including defacing operations to remove facial features. This study evaluates the impact of defacing on AI-driven organ segmentation in head-and-neck (HN) computed tomography (CT) images.

**Methods:**

Two defacing algorithms, DeIdentifier and *mri_reface*_0.3.3, were applied to 50 patient CT scans. Segmentation accuracy was assessed using two commercially available AI segmentation tools, INTContour and AccuContour^®^, and evaluated using Dice similarity coefficient (DSC), Hausdorff Distance at the 95th percentile (HD95), and Surface Dice Similarity Coefficients (SDSC) with 2 mm tolerance. Dose differences (D0.01cc) were calculated for each structure to evaluate potential clinical implications. Statistical comparisons were made using paired t-tests (p<0.05).

**Results:**

The results showed that defacing significantly impacted segmentation of on-face structures (e.g., oral cavity, eyes, lacrimal glands) with reduced DSC (<0.9) and higher HD95 (>2.5 mm), while off-face structures (e.g., brainstem, spinal cord) remained largely unaffected (DSC >0.9, HD95 <2 mm). DeIdentifier better preserved Hounsfield Units (HU) and anatomical consistency than *mri_reface*, which introduced more variability, including HU shifts in air regions. Minor differences in segmentation accuracy were observed between defacing algorithms, with mri_reface showing slightly greater variability. AccuContour showed slightly greater segmentation variability than INTContour, particularly for small or complex structures. Dose distribution analysis revealed minimal differences (<20 cGy) in most structures, with the largest variation observed in the Brainstem (34 cGy), followed by Lips_NRG (28 cGy) and Brain (25 cGy).

**Conclusion:**

These findings suggest that while defacing alters segmentation accuracy in on-face regions, its overall impact on off-face structures and radiation therapy planning is minimal. Future work should explore domain adaptation techniques to improve model robustness across defaced and non-defaced datasets, ensuring privacy while maintaining segmentation integrity.

## 1 Introduction

Data sharing is critical for advancing research and enabling multi-institutional collaborations, particularly in fields like radiation oncology where diverse datasets are essential for robust and generalizable findings. With the rise of artificial intelligence (AI) in medical applications, the demand for large, diverse, and high-quality datasets has grown exponentially. AI has demonstrated remarkable success in analyzing imaging data, such as tumor detection and organ segmentation, driving a need for shared medical image datasets to improve model performance and reproducibility (1). Initiatives like The Cancer Imaging Archive (TCIA) provide access to a wide range of anonymized datasets, supporting research in cancer diagnostics, treatment planning, and medical imaging analysis (2).

However, medical imaging data contain sensitive patient information, which raises privacy concerns. The Health Insurance Portability and Accountability Act (HIPAA) mandates the removal of protected health information (PHI) before sharing, except in specific circumstances or with the patient’s written authorization. Identifiers such as names and phone numbers are explicitly listed in HIPAA as PHI that must be removed (3). To comply with these regulations, tools have been developed to de-identify medical images by stripping Digital Imaging and Communications in Medicine (DICOM) files of PHI-containing tags or keeping only raw image pixels in formats such as Neuroimaging Informatics Technology Initiative (NIfTI) and nearly raw raster data (NRRD) (4, 5).

Despite these efforts, scans of the head pose unique challenges, as 3D renderings of the face can potentially re-identify subjects even after explicit PHI attributes are removed (6). This raises ethical concerns about balancing open data sharing with patient privacy. As a result, specialized tools have been developed to remove facial features from medical images to enhance privacy and facilitate safe data sharing (7–16). Addressing these challenges is crucial for ensuring the integrity and utility of shared datasets in advancing AI applications in radiation therapy.

The alteration of medical image data, such as through defacing operations, poses significant challenges to its usability, particularly when the data is applied beyond its original purpose. Defacing, while necessary for patient privacy protection, can inadvertently impact the integrity of the data in ways that compromise downstream applications. Studies have demonstrated that radiomics metrics, which are often used to quantify features within medical images, are particularly sensitive to changes in the facial region, with significant deviations observed following defacing operations (17, 18). Recent work has also examined how defacing affects the reliability of deep learning-based segmentation models in head-and-neck imaging. A study using magnetic resonance imaging (MRI) data demonstrated that applying common defacing algorithms significantly reduced segmentation accuracy for organs at risk, especially when models were trained on original images but evaluated on defaced ones (19). These findings highlight that privacy-preserving preprocessing methods may compromise the quality of AI outputs in radiotherapy applications if not carefully validated (19). However, a key question is whether defacing unintentionally impacts regions beyond the face. While current studies suggest that unaffected regions, like the brain, generally retain sufficient quality for traditional clinical and research purposes (6, 20), further validation is needed to assess whether the performance of convolutional neural network (CNN) based AI models on unaffected regions would be affected.

AI models have shown a pronounced vulnerability to image manipulation. It has been reported that even modification of a single pixel can drastically alter the output of a state-of-the-art models, leading to significant errors in interpretation and decision-making (21). Such vulnerabilities emphasize the importance of evaluating how defacing operations impact AI performance, not just in the defaced regions but also in untouched areas. This is particularly relevant in the context of organ segmentation models used in radiation therapy, where accuracy is paramount. Small inaccuracies in target or organ-at-risks can lead to either inadequate tumor coverage or un-necessary normal tissue toxicity (22–24).

It is hypothesized that defacing operations, by modifying part of the input data, could unexpectedly affect an AI model’s ability to accurately interpret even unaffected regions. Furthermore, such alterations may degrade the generalizability and robustness of these models when applied across datasets with varying degrees of defacing (22). This study specifically focuses on understanding the implications of defacing operations on organ segmentation AI models used in radiation therapy, with an emphasis on determining whether segmentation accuracy is preserved in regions outside the facial area, such as the brain or other critical organs.

## 2 Methods

This study used head-and-neck (HN) computed tomography (CT) data from 50 randomly selected patients (mean age 63.5 ± 19.9 years; 24 females, 26 males) treated at a single institution. Ethical approval was obtained from the Mayo Clinic Institutional Review Board (IRB). All patients were treated with photon-based volumetric modulated arc therapy (VMAT) plans, created using the Varian Eclipse treatment planning system (Varian Medical Systems, Palo Alto, CA). The dataset includes patients both with and without dental artifacts, as these were not excluded during cohort selection. Of the 50 patients, 44 had iMAR enabled. Among the remaining 6 patients, 4 exhibited visible streaks due to dental artifacts, while 2 had no dental artifacts. Organs-at-risk (OARs) were delineated by commercial segmentation tools. In cases where multiple guideline-based variants were available for the same structure, the vendor explicitly included suffixes (e.g., “_NRG”) to indicate that the contour followed NRG Oncology consensus recommendations (25). These labels were directly generated by the software and were not manually modified.

### 2.1 Defacing algorithms

In this study, two defacing algorithms, DeIdentifier and *mri_reface*, were used to remove or obscure facial features. DeIdentifier (Carina AI, Lexington, Kentucky, USA) is a commercial software that includes the functions of removing Protected Health Information (PHI) from DICOM metadata, randomly shifting dates while retaining temporal relationships, and using natural language processing (NLP) to redact burned-in PHI from image overlays and reports. It also has a defacing operation. For defacing, it uses an artificial intelligence method to detect the face and applies a generated mask to obscure facial features while preserving critical head and neck regions for research. It supports both DICOM and NIFTI formats and provides a Graphical user interface (GUI) as well as command line tool for seamless workflow integration.

The *mri_reface_0.3.3* algorithm (13) replaces identifiable facial features in MRI and CT scans with an average face template. It aligns scans to a standardized template using image registration techniques and substitutes facial regions with template features. This approach removes facial features of an individual subject while preserving anatomical context and minimizing artifacts that could interfere with brain measurement software. *mri_reface* is provided as a compiled Matlab application that requires Linux with the Matlab runtime installed. The tool is provided without GUI.

All original HN CT scans were in DICOM format and were used as inputs for both DeIdentifier and *mri_reface*. DeIdentifier outputted a de-identified and defaced HN CT in DICOM format. For *mri_reface*, a separate open-source NIFTI-to-DICOM converter (nifti2dicom) (26) was used to convert the defaced NIFTI file back to DICOM for comparison and analysis.

### 2.2 Segmentation models

In this study, two commercially available, FDA-approved auto-segmentation software solutions were used for HN structure segmentation: INTContour by CARINA AI (Carina AI, Lexington, Kentucky, USA) and AccuContour^®^ by Manteia Technologies (Manteia Medical Technologies Co., Milwaukee, Wisconsin, USA). Although the supported structures differ, there are 22 common key HN structures between the two segmentation software, providing a basis for direct comparison of the performance change due to defacing.

### 2.3 Experimental workflow

This study was conducted in a series of steps to evaluate the impact of defacing algorithms on segmentation performance, focusing on both the effect of different defacing methods with the same auto-segmentation software and the effect of defacing across different auto-segmentation platforms:

#### 2.3.1 Impact of different defacing algorithms evaluated using the same auto-segmentation software (INTContour)

Fifty HN CT images were defaced using two defacing platforms: DeIdentifier and *mri_reface*. Each set of defaced images, along with the original HN CT images, was segmented using the INTContour platform. The segmentation results were saved as DICOM radiotherapy structure (RT Structure) files. The segmentations generated from the original and defaced images were compared to assess the impact of defacing on segmentation accuracy.

#### 2.3.2 Impact of defacing algorithms across different auto-segmentation software

To compare the impact of defacing across different auto-segmentation software, the same set of 50 HN CT images defaced with DeIdentifier was segmented using both INTContour and AccuContour^®^. The segmentation outputs were compared with the segmentations performed on the original HN CT images using each respective platform to evaluate the impact of defacing. Since the supported structures vary between the two auto-segmentation software, the comparison was limited to the 22 structures that are supported by both software.

To assess the impact of defacing based on anatomical location, we categorized all structures into on-face and off-face groups according to their proximity to the defaced facial surface. Specifically, on-face structures were defined as superficial structures located within approximately 5 mm of the outer facial surface and thus most likely to be directly affected by defacing (e.g., eyes, lenses, oral cavity). Off-face structures were deeper or posterior and therefore less likely to be modified (e.g., brain, brainstem, spinal cord). Although the oral cavity is partially internal, it frequently overlaps with defaced regions in anterior CT slices and was classified as on-face based on visual confirmation of masking effects. Additionally, we identified which structures were supported by both AI segmentation platforms (INTContour and AccuContour) and which were unique to AccuContour. **Table 1** summarizes this classification and platform availability across all evaluated structures.

**Table 1:** Categorization of on-face and off-face structures and platform availability.

On-face structures	Off-face structures	Accucontour-only structures
EYE_L	**BONE_MANDIBLE**	TRACHEA
EYE_R	BRACHIALPLEX_L	SUB_MANDIB_L
LENS_L	BRACHIALPLEX_R	SUB_MANDIB_R
LENS_R	CEREBELLUM_L	HIPPOCAMPUS_L
LIPS_NRG	CEREBELLUM_R	HIPPOCAMPUS_R
GLND_LACRIMAL_L	CERVICALSPINE	EXT_AUD_CANAL_L
GLND_LACRIMAL_R	**COCHLEA_L**	EXT_AUD_CANAL_R
CAVITY_ORAL	**COCHLEA_R**	NASAL_CAVITY
CAVITY_ORAL_NRG	**BRAIN**	SEMI_CIR_CANAL_L
	**BRAINSTEM**	SEMI_CIR_CANAL_R
	ESOPHAGUS	MASTOID_L
	GLND_SUBMAND_L	MASTOID_R
	GLND_SUBMAND_R	
	GLOTTIS_NRG	
	INNEREAR_L	
	INNEREAR_R	
	JOINT_TM_L	
	JOINT_TM_R	
	LN_NECK_III_L	
	LN_NECK_III_R	
	LN_NECK_II_L	
	LN_NECK_II_R	
	LN_NECK_IVa_L	
	LN_NECK_IVa_R	
	LN_NECK_Ib_L	
	LN_NECK_Ib_R	
	**LARYNX_NRG**	
	LARYNX	
	LARYNX_SG_NRG	
	**LOBE_TEMPORAL_L**	
	**LOBE_TEMPORAL_R**	
	MIDEAR_L	
	MIDEAR_R	
	MUSC_CRICOPHAR	
	**OPTICCHIASM**	
	**OPTICNRV_L**	
	**OPTICNRV_R**	
	PCM_I	
	PCM_M	
	PCM_S	
	**PAROTID_L**	
	**PAROTID_R**	
	PHARYNX	
	**PITUITARY**	
	**SPINALCORD**	
	THORACICSPINE	
	**THYROID**	

Structures were grouped based on anatomical proximity to the defaced region. On-face structures include those located at or near the anterior facial surface, which are most directly impacted by defacing algorithms. Off-face structures are anatomically distant and generally unaffected by defacing. The 22 structures evaluated by both INTContour and AccuContour are shown in bold. An additional set of structures available only through AccuContour are shown in the third column.

### 2.4 Evaluation metrics

To evaluate the extent of image manipulation introduced by defacing, we computed voxel-wise Hounsfield Unit (HU) differences (ΔHU) between the original and defaced CT images for all 50 head-and-neck cases. We first verified that the defacing process preserved alignment; rigid registration was then applied only as a safeguard against occasional orientation inconsistencies from NIfTI to DICOM conversions (e.g., a 180° anterior–posterior flip in *mri_reface*). Voxel-wise subtraction was then performed to generate ΔHU maps, which were used to visualize the spatial extent and magnitude of intensity changes introduced by defacing. For each case, we analyzed ΔHU distributions across facial and cranial regions, with particular attention to air voxels, soft tissues, and bony structures. The impacted depth was defined as the anterior–posterior distance from the facial surface to the deepest visibly altered voxel and was summarized across patients to compare the anatomical reach of defacing between algorithms.

To evaluate the impact of defacing algorithms on AI segmentation, various metrics were calculated between the segmentations on defaced and original images, including the Dice coefficient, Hausdorff Distance at the 95th percentile (HD95), and Surface Dice Similarity Coefficients (SDSC) with 2 mm tolerance. The Dice coefficient measures the spatial overlap between the segmented and ground-truth regions, providing an overall agreement score. HD95 assesses boundary alignment by calculating the maximum distance at which 95% of points on the segmented surface lie within the ground-truth region and vice versa. SDSC (2mm) quantifies agreement of two structure surfaces within 2mm tolerance thresholds.

In the context of radiation therapy, anatomical structures guide both target optimization and avoidance during treatment planning. Any changes in segmentation may lead to variations in dose distribution, affecting treatment optimization and evaluation. To assess this, we computed the D0.01cc metric for each structure, defined as the minimum dose received by the hottest 0.01 cc of tissue (approximately 10 mm³). This metric is clinically relevant as it captures localized dose hotspots, which are especially important in small or radiosensitive organs and is sensitive to small segmentation changes.

### 2.5 Statistical analysis

Paired t-tests were used to compare segmentation metrics (Dice, HD95, SDSC 2mm) between two defacing algorithms; DeIdentifier and *mri_reface*. A significance level of p < 0.05 was applied.

## 3 Results

**Figure 1:**
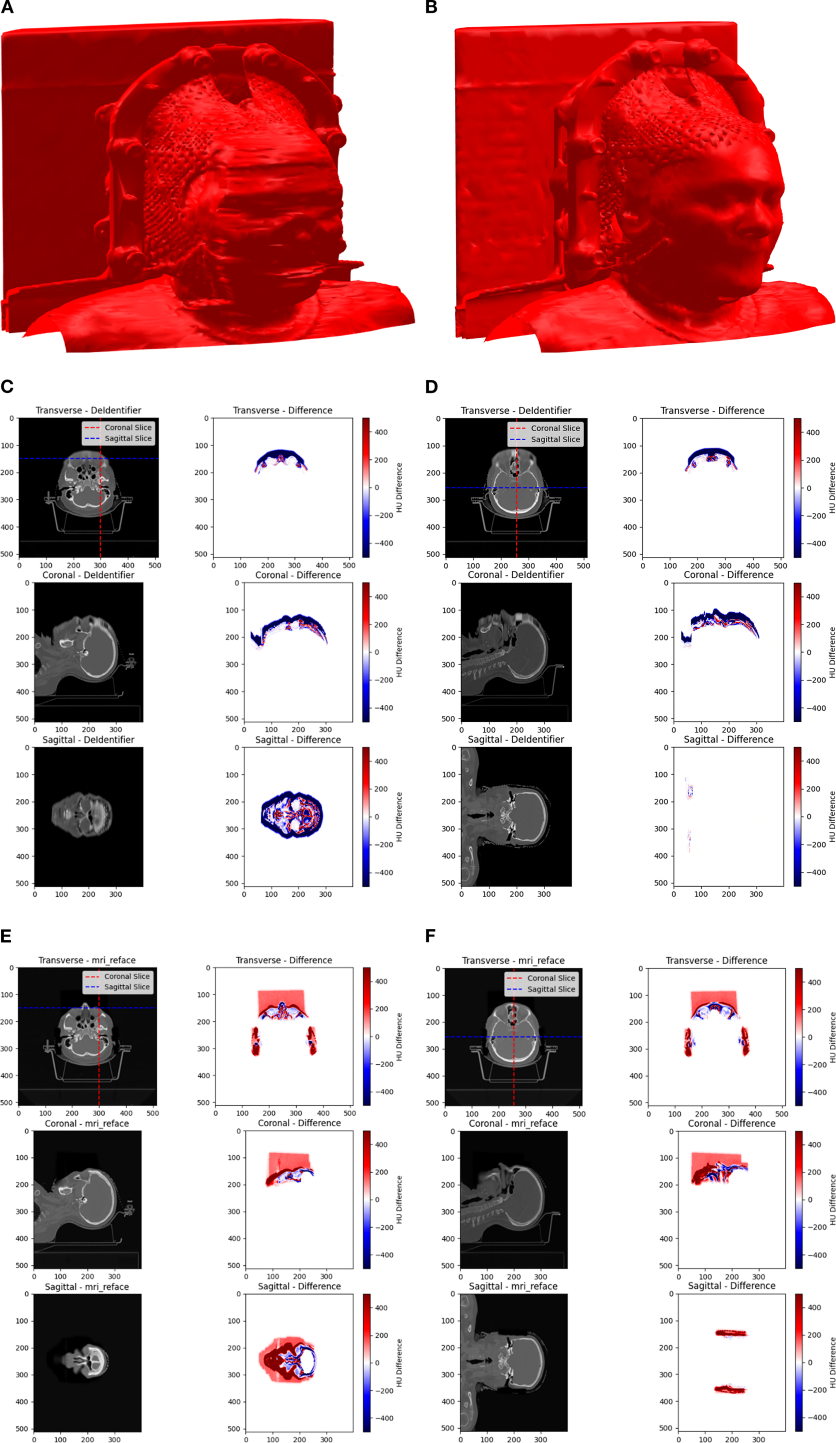
A 3D rendering of an example HN CT after defacing using **(A)** DeIdentifier and **(B)** *mri_reface*. Comparison of Hounsfield Unit (HU) distributions and differences across transverse, coronal, and sagittal planes between the original and defaced CT scans is shown for DeIdentifier with **(C)** slices near the front of the face and **(D)** middle slices in all directions, and for *mri_reface* with **(E)** slices near the front of the face and **(F)** middle slices in all directions. Warmer colors (red) represent positive differences, while cooler colors (blue) represent negative differences. The images highlight the spatial variability in HU modifications introduced by the defacing process.

The extent of image manipulation for both DeIdentifier and *mri_reface* using HN CT images was assessed. As can be seen in the 3D rendering of the surface of an example HN CT after defacing for both DeIdentifier (**Figure 1A**) and *mri_reface* (**Figure 1B**). DeIdentifier applied a mask over the facial region, introducing an added surface layer while preserving deeper anatomical structures. This masking primarily affected superficial regions. The added layer has a randomized blend in with the facial region so that it is impossible to restore the original face through thresholding or a morphological operation. Since *mri_reface* replaces the entire facial region with an average template, the defaced image retains a human-like appearance but lacks distinctive facial features. A previous study has demonstrated that *mri_reface* effectively removes identifiable facial structures, significantly reducing reidentification risks (16).

Hounsfield Unit (HU) differences between the original and defaced images were calculated voxel-wise for all 50 patients to visualize and quantify localized effects of defacing. ΔHU maps revealed that both algorithms predominantly altered facial regions, including the eyes, lacrimal glands, lenses, optic nerves, nasal cavity, and lips, while ΔHU ≈ 0 was observed in deeper anatomical structures. Across patients, the median HU change in air voxels remained 0 for DeIdentifier but deviated to −90 HU for *mri_reface* (mean air HU = −1090 vs. −1000 in original). These spatial differences are illustrated in the representative ΔHU maps (**Figures 1C–F**). For *mri_reface*, this HU shift is associated with a rectangular cuboid region visible anterior to the patient (**Figures 1E, F**). This artifact arises from insertion of the average face template, which extends slightly beyond the patient contour and alters the expected air voxel values, whereas DeIdentifier preserved air voxels near −1000 HU. In addition to intensity shifts, we quantified the impacted depth and found that *mri_reface* consistently reached deeper anatomical regions (mean depth: 75 mm) compared to DeIdentifier (mean depth: 45 mm), supporting its broader area of manipulation.

**Figure 2:**
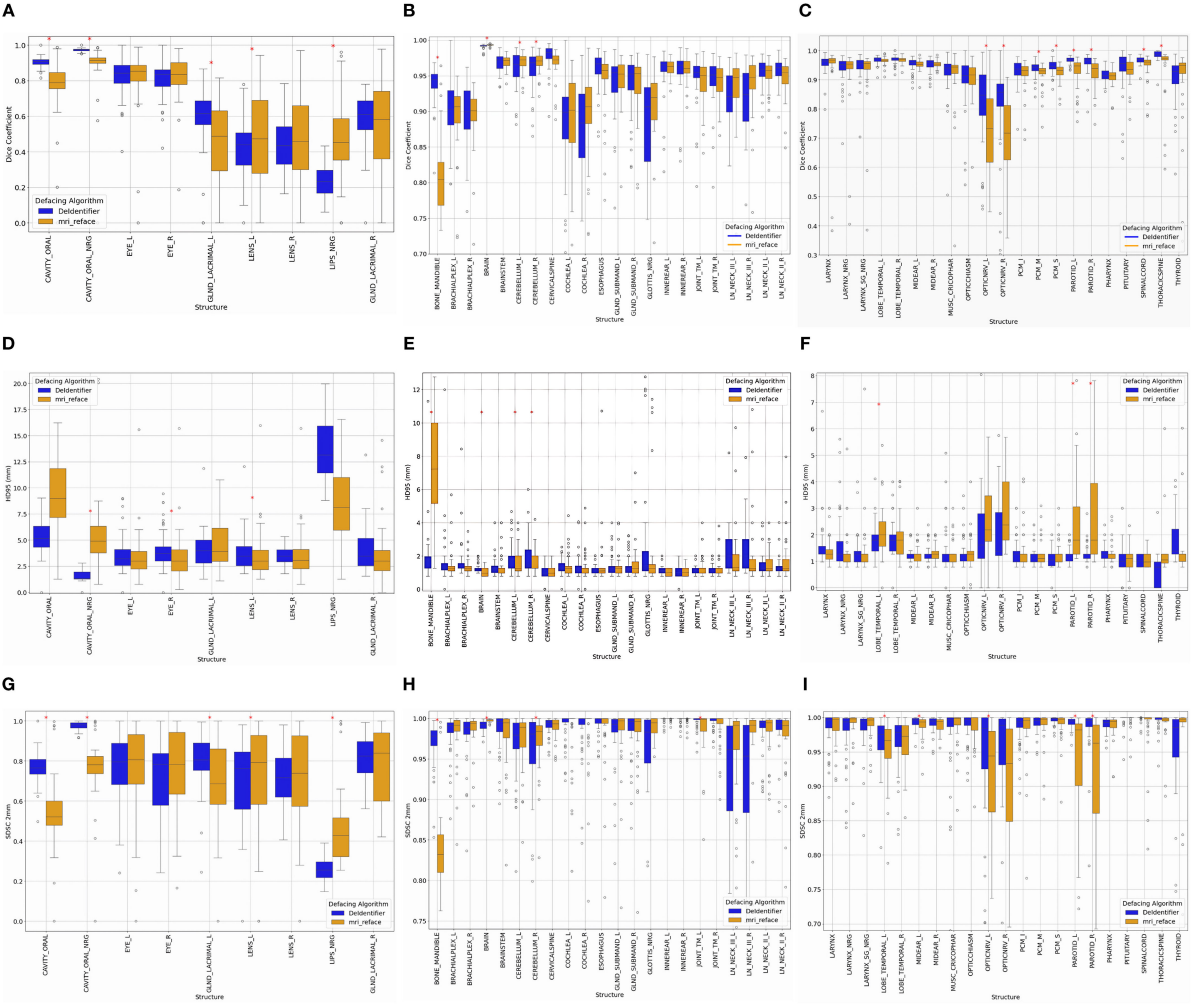
Impact of defacing on segmentation, comparing De-Identifier and *mri_reface*. Changes in DSC, HD95, and SDSC (2 mm tolerance) were calculated for **(A, D, G)** structures directly impacted on the front face, **(B, E, H)** structures away from the front face, and **(C, F, I)** continued structures away from the front face. Statistically significant structures are indicated using a red asterisks (*) (p<0.05).

**Figure 3:**
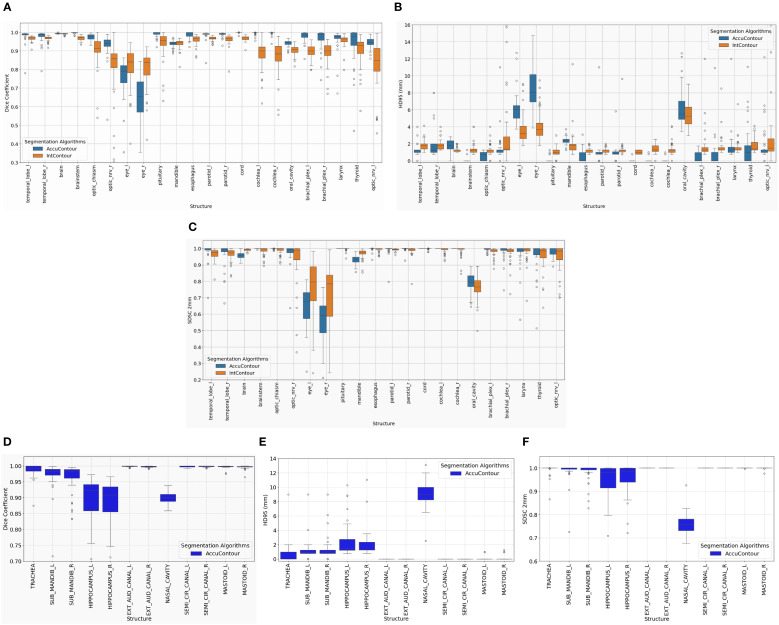
Performance of AccuContour^®^ segmentation using the De-Identifier algorithm. The boxplots show **(A)** Dice, **(B)** 95^th^ Percentile Hausdorff Distance, and **(C)** Surface Dice Similarity Coefficient within 2 mm for each common HN structure between INTContour and AccuContour. The remaining structures from AccuContour in the HN region were plotted, **(D)** Dice, **(E)** 95^th^ Percentile Hausdorff Distance, and **(F)** Surface Dice Similarity Coefficient within 2 mm.

While detailed metrics for every structure are shown in **Figures 2** and **3**, we highlight key representative values in the text to illustrate general trends across defacing algorithms and segmentation tools.

### 3.1 Impact from different defacing algorithms

Both defacing algorithms demonstrate the expected impact on segmentation accuracy, with variations depending on the anatomical location and proximity to the defacing modifications. Structures can be broadly categorized into on-face and off-face groups (**Table 1**), which show distinct segmentation performance patterns.

On-face structures, such as the oral cavity, eyes, lenses, and lacrimal glands (**Figures 2A, D, G**), experience greater segmentation variability due to their direct proximity to defacing modifications. In general, on-face structures exhibit lower DSC (< 0.9), higher HD95 values (>2.5 mm), and lower SDSC_2mm values (<0.9), reflecting reduced segmentation accuracy, boundary precision, and surface alignment. For example, both methods showed an impact in the oral cavity, with the median Dice coefficient for *mri_reface* at 0.79 and for DeIdentifier at 0.91, indicating some degree of alteration in both cases. Similarly, median HD95 values for the lacrimal glands (right) are 3 mm for *mri_reface* and 3.5 mm for DeIdentifier, while SDSC_2mm values for the lenses (right) are 0.74 for *mri_reface* and 0.72 for DeIdentifier. Although DeIdentifier generally demonstrates higher consistency and better preservation of anatomical integrity in these regions, the overall differences remain within a relatively small range for most structures.

While on-face structures show significant segmentation variability, off-face structures remain largely stable, experiencing minimal segmentation inconsistencies. Off-face structures, such as the brain, cerebellum, brainstem, spinal cord, and cervical spine (**Figures 2B, C, E, F, H, I**), exhibit high segmentation accuracy, with Dice scores typically exceeding 0.9, low HD95 values (<2 mm), and high SDSC_2mm values (>0.97). For example, HD95 values for the brainstem are 1.3 mm for both DeIdentifier and *mri_reface*, while SDSC_2mm values remain consistently high (>0.95) for both algorithms.

For smaller, detailed structures—such as the cochlea and brachial plexus—segmentation metrics require additional consideration. These structures naturally tend to exhibit lower Dice coefficients due to their small volume, making them highly sensitive to segmentation differences. For example, the median Dice coefficients for the cochlea were 0.9 and 0.91 (left and right, respectively) for *mri_reface*, compared to 0.89 and 0.88 for DeIdentifier. However, the low HD95 values (1–2 mm for both algorithms) and high SDSC_2mm values (~1, representing minimal change beyond 2 mm) suggest consistent boundary alignment, indicating that these regions are minimally impacted by defacing. This observation underscores the importance of interpreting Dice coefficients for small structures alongside HD95 values, which often provide clearer insight into boundary precision and the true impact of defacing.

While both algorithms perform well in most of the off-face structures, *mri_reface* exhibits slightly greater variability and more outliers compared to DeIdentifier, suggesting minor segmentation inconsistencies even in areas not directly impacted by defacing. This trend reinforces that face structures are significantly impacted by defacing, while off-face structures experience less variation, aside from a few artifacts due to small size.

There are a few structures that are more noticeably affected by the choice of defacing algorithm. For example, *mri_reface* has a greater impact on segmentation accuracy in structures like the optic nerve (median Dice coefficient 0.72 vs. 0.85 for DeIdentifier), parotid gland, and mandible. Meanwhile, DeIdentifier shows more variable performance in structures like LN_Neck_III.

### 3.2 Impact on different AI segmentation algorithms

The effect of defacing operation on AccuContour segmentation was similar to INTContour in that both on-face and off-face structures were affected, with the larger impact on on-face structures. However, each AI segmentation algorithm demonstrates slightly different levels of robustness depending on the structure.

As summarized in **Table 1**, 22 common structures were supported by both segmentation platforms, while an additional 12 structures were exclusive to AccuContour. **Figure 3A–C** shows segmentation accuracy across the 22 shared structures, and **Figures 3D, E** presents results for the AccuContour-only structures. AccuContour exhibits variable performance across metrics, with segmentation accuracy differing by anatomical region. In on-face regions, such as the optic nerves, optic chiasm, and eyes, AccuContour shows lower Dice coefficients, higher HD95 values, and variable SDSC (2mm) scores, indicating challenges in boundary precision and volumetric overlap. For off-face structures, including the brainstem, temporal lobes, and mandible, AccuContour demonstrates higher Dice coefficients and more stable HD95 and SDSC (2 mm) values. Several structures appear largely unaffected by defacing, maintaining consistent segmentation quality. INTContour, by contrast, demonstrates a more consistent performance across all anatomical regions.

**Figure 4:**
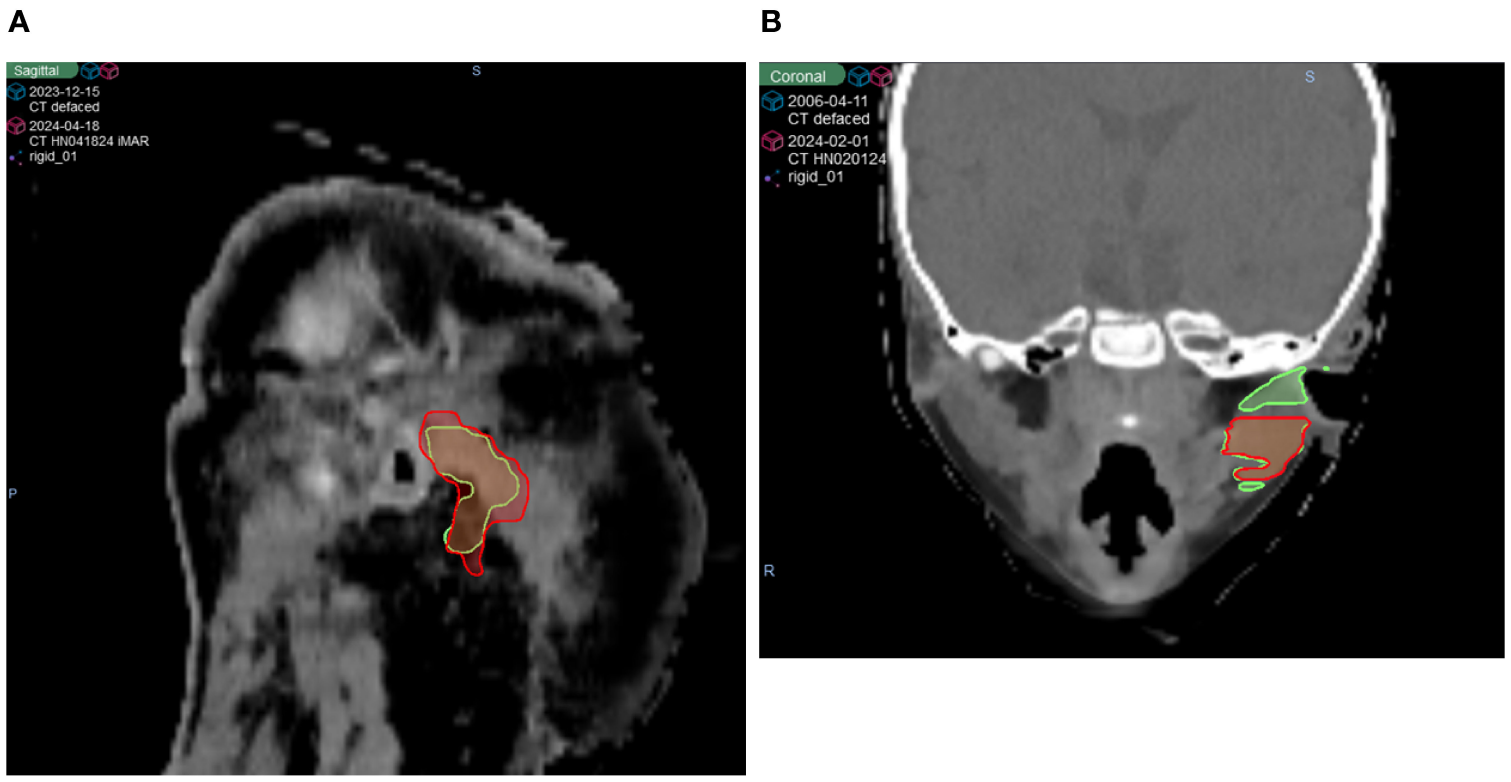
Two patient cases illustrating poor performance by both AI segmentation tools. The original (green) and defaced (red) contours are overlaid on the defaced CT. **(A)** The right parotid segmented using INTContour, with a Dice score of 0.79, HD95 of 9.58 mm, and SDSC_2mm of 0.78. **(B)** The left parotid segmented using AccuContour, with a Dice score of 0.83, HD95 of 11 mm, and SDSC_2mm of 0.80.

As shown in **Figures 3A–C**, while many structures demonstrated strong overall robustness to the defacing operation—achieving >0.90 DSC, >0.95 SDSC_2mm, and <2 mm HD95 in most cases—some outliers were notably affected. One such case is illustrated in **Figure 4**, where the Parotid_R was segmented using INTContour (**Figure 4A**) and the Parotid_L using AccuContour (**Figure 4B**). These two cases exhibited a large discrepancy in their HD95 values, with INTContour and AccuContour achieving HD95 measurements of 9.58 mm and 11 mm, respectively. This discrepancy underscores that while defacing does not significantly impact most structures, certain cases—particularly those involving larger or more complex anatomies like the mandible—may still experience notable segmentation variability.

**Figure 5:**
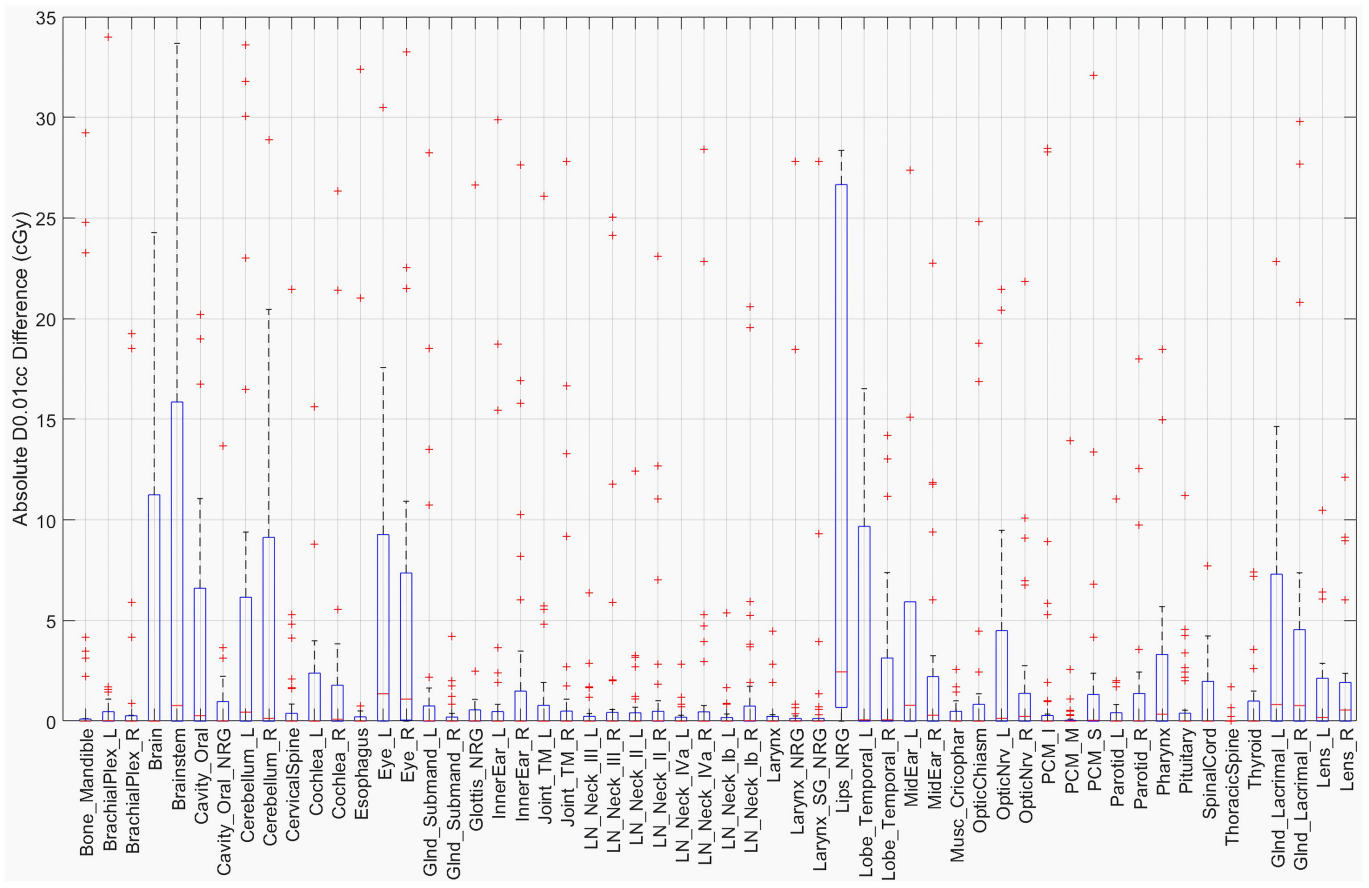
Absolute D0.01cc difference for each structure between the original and defaced images.

The D0.01cc dose was determined using the masked region for each structure in both the original and defaced HN cases. The absolute difference in centigray (cGy) is shown in **Figure 5**. Most structures demonstrated differences below 20 cGy, with the largest observed in the Brainstem (34 cGy), Lips_NRG (28 cGy), and Brain (25 cGy). These findings suggest that defacing had a limited effect on high-dose sub-volumes for the majority of structures.

## 4 Discussion

This study systematically evaluates the impact of defacing operations on AI organ segmentation in HN CT images. Our findings demonstrate that defacing alters AI segmentation outputs even for structures that are not directly modified by the defacing process. This effect is not limited to a specific defacing algorithm or a single auto-segmentation platform, indicating that any modification to input medical images, even in regions seemingly unrelated to target structures, can propagate downstream effects in automated segmentation models.

Despite these observed changes, the overall impact remains relatively small. While certain structures exhibited notable decreases in DSC, the HD95 and SDSC at 2 mm tolerance indicate that boundary misalignment remains minimal in most cases. However, a few outlier cases, as seen in **Figure 3**, exhibited HD95 values exceeding 10 mm, suggesting localized segmentation disruptions. Interestingly, these larger segmentation discrepancies did not always translate into significant changes in D0.01cc dose. This may be because the affected structures in these cases were located farther from high-dose target regions, reducing the clinical impact. Supporting this, the D0.01cc difference analysis showed that most structures experienced differences under 20 cGy, with the exception of the Brainstem (34 cGy), Lips_NRG (28 cGy), and Brain (25 cGy), suggesting that defacing generally preserves high-dose information within clinically relevant tolerance. These findings suggest that, while defacing influences segmentation accuracy, its overall clinical impact on treatment planning is likely limited and depends on the spatial relationship between affected structures and high-dose regions.

Although *mri_reface* is designed to alter only the facial region, segmentation models analyze the entire image volume holistically. Thus, changes introduced in one area, such as the face, can influence predictions in distant, off-face regions. These indirect effects are likely model-driven rather than caused by direct HU changes in those areas, underscoring the sensitivity of AI-based segmentation to global image context.

Both INTContour and AccuContour are FDA-cleared tools that perform well in routine clinical use, with high accuracy reported for non-defaced CT images. Our analysis was not designed to directly compare their baseline performance, but rather to examine how each responds to image modifications introduced by defacing. The greater variability observed for AccuContour in the defaced setting therefore reflects differences in robustness to defacing, rather than underlying disparities in their performance on standard CT scans.

A crucial consideration is whether defacing introduces systematic bias in AI-driven segmentation. Our findings suggest that defacing is a viable approach for preserving privacy while maintaining segmentation accuracy for most structures, making it a reasonable preprocessing step before sharing CT images that include the head region. However, a potential challenge arises when AI models trained on defaced images are deployed in clinical practice, where non-defaced images are typically used. Since defacing alters only specific regions, AI models may develop an unintended dependence on these modifications, leading to systematic under- or over-segmentation of structures adjacent to the defaced region. Future research should investigate whether fine-tuning AI models on defaced images enhances their robustness and ensures consistent performance across both defaced and non-defaced datasets.

In radiation oncology, defacing must preserve critical quantitative information to ensure the integrity of segmentation and dosimetric accuracy. Previous studies have shown that certain defacing methods, such as quickshear and CT_biometric_mask, introduced variability in key structures, including the clinical target volume (CTV) and gross tumor volume (GTV) (27). In contrast, DeIdentifier demonstrated better preservation of radiomic features and dosimetric accuracy by masking facial structures without altering surrounding tissues. Given these findings, our study focused on evaluating two defacing methods, DeIdentifier and *mri_reface*, both of which have been shown to be less invasive. Our results indicate that the differences between these two methods were minimal, reinforcing that appropriately selecting defacing techniques can maintain segmentation accuracy while protecting patient privacy.

These findings contrast with those of Sahlsten et al. (19), who demonstrated substantial degradation in segmentation accuracy when applying common defacing tools to MRI head-and-neck datasets, particularly when models trained on original data were tested on defaced images. Their results were based on experimental 3D U-Net models and non-clinical defacing methods and showed that several algorithms either failed to deface CTs or removed critical anatomical voxels (19). In contrast, our study, which focused on CT data and used two FDA-cleared commercial segmentation tools, suggests that these platforms may be more robust to defacing, especially when defacing algorithms are carefully selected.

Data sharing is essential for advancing research, as it allows investigators to analyze medical imaging data in novel ways beyond the original intent. A notable example is Thor et al. (28), who demonstrated that re-segmenting and re-analyzing dosimetry led to stronger predictors of survival based on three heart dose metrics. In addition to improving contouring consistency through re-segmentation, AI models could further enhance research by segmenting structures that were previously overlooked, such as swallowing muscles and cardiac substructures, enabling new associations between dose metrics and clinical outcomes. Defacing plays a critical role in facilitating the sharing of medical images by addressing privacy concerns. Our study suggests that defacing is unlikely to compromise the re-segmentation process or the subsequent re-analysis of dose-volume histograms, reinforcing its viability for research and clinical applications.

The dataset included 50 patients, of whom 44 had iMAR enabled. Of the remaining six patients, four exhibited visible streaks due to dental artifacts, and two had none. Although this study did not present results stratified by artifact status, we reviewed the segmentation performance across patients with and without dental artifacts and found minimal differences for non-facial structures. On-face structures such as the lips and oral cavity, known to be directly impacted by defacing, showed greater variability. These structures would not typically be used in clinical or research analyses involving defaced images and would therefore be excluded from downstream evaluations. The overall trends reported in this study remained consistent regardless of artifact presence.

Moreover, emerging areas of research in HN radiation therapy highlight the need to segment additional structures that were not included in the original dataset. Recent studies have emphasized the delineation of swallowing and chewing-related organs to refine dose tolerance limits and optimize treatment planning. AI segmentation models have been developed to automatically contour these structures (29, 30). The ability to retrospectively apply these models to existing clinical trial data has significant implications for evaluating dose metrics and tolerance thresholds. Additionally, studies have investigated the potential of using auto-segmentation to reduce contouring variability and dose inconsistency in clinical trials (28). However, when using defaced images in such studies, it is crucial to assess how segmentation accuracy and subsequent dose evaluations are influenced by the defacing process. This includes evaluating performance in the presence of artifacts, such as dental fillings, which may amplify segmentation variability but appeared to have limited overall impact in our study.

While our results indicate that defacing does not substantially impact segmentation for most structures, the presence of a few outlier cases suggests that certain structures may be more susceptible to segmentation variability. This raises concerns about whether AI segmentation models trained on non-defaced datasets can consistently generalize to defaced images. To address this, future research should evaluate whether incorporating defaced images into model training pipelines can enhance robustness. Additionally, domain adaptation techniques could help mitigate segmentation variability introduced by defacing, improving model consistency across different preprocessing conditions.

Although our findings suggest that the overall effect on segmentation accuracy and dosimetric evaluation is minimal, researchers must remain cognizant of potential inconsistencies, particularly when analyzing newly segmented structures or applying AI models retrospectively. Future work should focus on refining defacing algorithms to minimize alterations in medically relevant regions while ensuring regulatory compliance and patient privacy.

This study has several limitations. It was conducted at a single institution using a curated dataset of 50 head-and-neck (HN) patients and evaluated two defacing algorithms, DeIdentifier (FDA-cleared) and *mri_reface*, and two commercial auto-segmentation tools. While these tools reflect clinically used systems, we acknowledge that segmentation performance and defacing effects may vary across institutions, imaging protocols, and software platforms. Therefore, the generalizability of our findings may be limited. However, the goal of this work is not to rank or endorse specific tools, but rather to highlight how commonly used defacing and segmentation algorithms can influence clinical imaging data. By providing a systematic analysis of segmentation and dosimetric variability introduced by defacing, this study aims to inform clinical and research users of potential pitfalls and best practices. Future multi-center studies incorporating a broader range of tools will be valuable to build on this foundational work and further validate these observations.

## Data Availability

The data analyzed in this study is subject to the following licenses/restrictions: Not available due to PHI and institutional policy. Requests to access these datasets should be directed to QC, Chen.Quan@mayo.edu.
